# Green Synthesis of Silver Nanoparticles with Antibacterial, Anti-Inflammatory, and Antioxidant Activity Using *Convolvulus arvensis*

**DOI:** 10.3390/ijms27031210

**Published:** 2026-01-25

**Authors:** Suzan Abdullah Al-Audah, Azzah Ibrahim Alghamdi, Sumayah I. Alsanie, Nadiyah M. Alabdalla, Amnah Alawdah, Norah Alenezi, Aisha AlShammari, Ibrahiem Taha, Ahmed Albarrag, Sumayah Aldakeel, Munirah Aldayel

**Affiliations:** 1 Department of Biology, College of Science, Basic and Applied Scientific Research Centre (BASRC), Imam Abdulrahman Bin Faisal University (IAU), P.O. Box 1982, Dammam 31441, Saudi Arabia; naalaudah@iau.edu.sa (S.A.A.-A.); azalghamdi@iau.edu.sa (A.I.A.); nmalabdallah@iau.edu.sa (N.M.A.); 2220040220@iau.edu.sa (A.A.); noalenzi@iau.edu.sa (N.A.); osalshammry@iau.edu.sa (A.A.); 2 Basic and Applied Scientific Research Center (BASRC), Imam Abdulrahman Bin Faisal University (IAU), P.O. Box 1982, Dammam 31441, Saudi Arabia; 3 Department of Internal Medicine, College of Medicine, King Fahd Hospital of the University, Imam Abdulrahman Bin Faisal University (IAU), P.O. Box 1982, Dammam 31441, Saudi Arabia; isaeed@iau.edu.sa; 4 Department of Pathology, College of Medicine, King Saud University and King Saud University Medical City, Riyadh 12354, Saudi Arabia; aalbarrag@ksu.edu.sa; 5 Genomic of Infectious Diseases Laboratory, Public Health Laboratory, Public Health Authority, Riyadh 13351, Saudi Arabia; sadakeel@pha.gov.sa; 6 Department of Biology, College of Science, King Faisal University (KFU), Al-Ahasa 31982, Saudi Arabia; maldayel@kfu.edu.sa

**Keywords:** antibiotic-resistant bacteria, *Escherichia coli*, *Staphylococcus aureus*, *Convolvulus arvensis*, antibacterial, anti-inflammatory, antioxidant, nanoparticles

## Abstract

Due to the indiscriminate use of antimicrobial drugs in the treatment of infectious diseases, human pathogenic bacteria have developed resistance to many commercially available antibiotics. Medicinal plants such as *Convolvulus arvensis* represent a renewable resource for the development of alternative therapeutic agents. This study aimed to evaluate the antibacterial activity of silver nanoparticles (AgNPs) biosynthesized from *C. arvensis* against two clinical antibiotic-resistant bacterial isolates. The pathogenic isolates were identified as *Staphylococcus aureus* MRSA and *Escherichia coli* ESBL using 16S rRNA gene sequencing. Silver nanoparticles were synthesized via a green synthesis approach, and their physicochemical properties were characterized using UV–Vis spectroscopy, scanning electron microscopy (SEM), Fourier transform infrared (FTIR) spectroscopy, zeta potential, and dynamic light scattering (DLS). The synthesized *C. arvensis*–AgNPs exhibited a surface plasmon resonance peak at 475 nm and predominantly spherical morphology with particle sizes ranging from 102.34 to 210.82 nm. FTIR analysis indicated the presence of O–H, C–O, C–N, C–H, and amide functional groups. The nanoparticles showed a zeta potential of −18.9 mV and an average hydrodynamic diameter of 63 nm. The antibacterial activity of the biosynthesized AgNPs was evaluated against methicillin-resistant *S. aureus* (MRSA and *ATCC 29213*) and *E. coli* (ESBL and *ATCC 25922*) using agar diffusion, minimum inhibitory concentration (MIC), and minimum bactericidal concentration (MBC) assays. Inhibition zones ranged from 10 to 13 mm, with MIC and MBC values of 12.5–25 µg/mL and 25–50 µg/mL, respectively. In addition, the nanoparticles exhibited antioxidant activity (DPPH assay, IC_50_ = 0.71 mg/mL) and anti-inflammatory effects as determined by protein denaturation inhibition. No cytotoxic effects were observed in the MCF-7 cell line at the MIC level. These findings suggest that *C. arvensis*–AgNPs have potential as natural antimicrobial, antioxidant, and anti-inflammatory agents.

## 1. Introduction

Globally, antibiotic resistance reduces the efficiency of conventional medications against numerous illnesses. According to the 2022 Global Antimicrobial Resistance and Use Surveillance System (GLASS) report, the resistance rates of several bacterial pathogens are alarmingly high. In 76 countries, the median incidences of third-generation cephalosporin-resistant *E. coli* at 42% and methicillin-resistant *Staphylococcus aureus* at 35% are major causes for concern [1].

One of the primary causes of hospital-acquired infections is the Gram-positive bacterium *Staphylococcus aureus*, which is capable of causing various skin infections and can spread throughout the body, leading to serious conditions such as wound infections, endocarditis, osteomyelitis, and toxic shock syndrome. It is also notoriously resistant to most antibiotics [2,3,4,5].

Similarly, *E. coli* is a widespread Gram-negative pathogen that can adapt to diverse environments through the acquisition of virulence and resistance factors. It poses a serious threat to public health, agriculture, and the food industry, particularly due to its high resistance to antibiotics such as ampicillin [6].

There are also other types of opportunistic bacteria, including *Streptococcus mutans*, a key contributor to dental caries, and *Pseudomonas aeruginosa*, whose resistance to antimicrobials is a global threat due to its ability to cause a wide range of serious hospital-acquired infections, highlighting the urgent need to develop novel antimicrobial agents that can complement or replace conventional antibiotics [7,8].

Many plant extracts are known for their uses as antimicrobials; for example, *Convolvulus arvensis* extract, with its long history in traditional medicine, has antibacterial, anti-inflammatory, and anti-fungal [9], and anti-cancer properties [10]. Other plant extracts, such as *Borassus flabellifer* root, showed antibacterial activity against four strains of bacteria; it reduced *S. aureus* and *E. coli* activity but needed to be used at higher concentrations to reduce the growth of *S. typhimurium* and *S. pneumoniae* [11]. According to [12], green tea extracts have bactericidal and inhibitory effects and can be used as a recognized treatment for a variety of bacterial infections, including *B. subtilis*, *S. aureus*, *P. aeruginosa*, and *E. coli.*

In response to the growing issue of antimicrobial resistance, nanotechnology has emerged as a promising alternative. Among the most widely used nanomaterials, silver nanoparticles (AgNPs) have demonstrated potent antimicrobial properties and are applied in both agricultural and medical fields [13]. Additionally, metal oxide nanoparticles have gained attention due to their unique physicochemical properties and broad biological applications, highlighting the growing importance of nanomaterials in addressing diverse biological and environmental challenges [14].

Various medicinal plants such as *Azadirachta indica*, *Camellia sinensis*, and *Moringa oleifera* have been extensively explored for the green synthesis of AgNPs due to their rich phytochemical content [15]. Moreover, plant extracts such as *Pluchea indica* leaf extract have been successfully used for the green synthesis of bimetallic nanoparticles, showing significant antibacterial activity against *E. coli*, *Pseudomonas aeruginosa*, *Staphylococcus aureus*, and *Bacillus subtilis*, highlighting the potential of plant-mediated nanomaterials in antimicrobial applications [16].

The green synthesis of AgNPs using plant-based biomolecules as reducing and stabilizing agents has gained considerable attention because of its eco-friendliness, low toxicity, and cost-effectiveness [17]. Recent studies have demonstrated that plant-mediated AgNPs exhibit well-defined crystalline structures, pronounced surface plasmon resonance, and effective antimicrobial activity against both Gram-positive and Gram-negative bacteria, highlighting their potential as efficient antimicrobial agents [18]. In addition to their established medical value, AgNPs have recently attracted attention for applications in food preservation, packaging, and pharmaceutical formulations owing to their strong antimicrobial and antioxidant activities, contributing to enhanced food safety, prolonged shelf life, and improved drug delivery efficiency [19,20,21,22]. The antimicrobial, antioxidant, and anti-inflammatory properties of AgNPs have been well documented in the literature [23,24]. Reduced silver exhibits toxicity toward microorganisms by disrupting cell walls and interfering with essential cellular functions through interactions of Ag ions with macromolecules, including inhibition of protein synthesis and alteration of cell membrane permeability, ultimately leading to cell death [25].

Although the green synthesis of silver nanoparticles using *Convolvulus arvensis* has been worked on in some studies, a comprehensive evaluation of their biological activities in a single study has not yet been performed. Therefore, the present study aims to provide an assessment of *C. arvensis*-derived silver nanoparticles, including their antibacterial activity against drug-resistant *Staphylococcus aureus* and *Escherichia coli*, as well as their antioxidant, anti-inflammatory, and cytotoxic properties.

## 2. Results

### 2.1. Bacterial Samples and Identification

**Figure 1:**
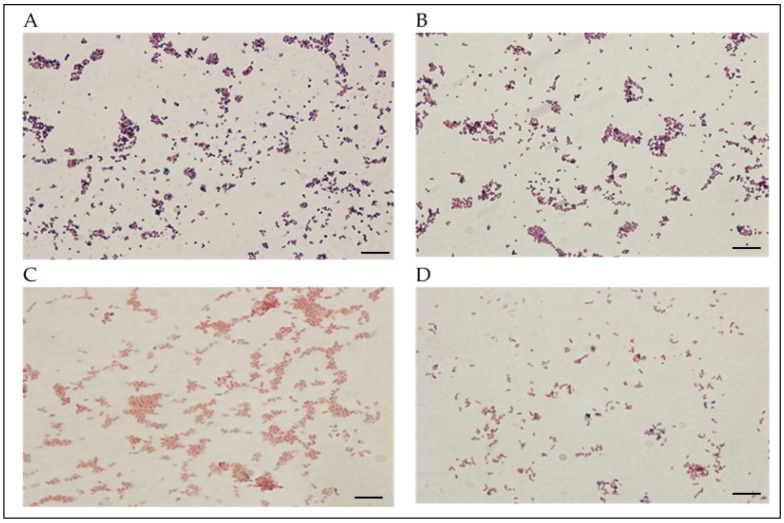
Microscopic images of Gram staining; positive Gram staining shows clustered bluish-purple cocci of (**A**) SP 1 strain and (**B**) *S. aureus* (ST), negative Gram staining shows pink short rods of (**C**) SP 2 strain and (**D**) *E. coli* (ST); light microscope, 100× magnification. Scale bars: 5µm.

The two clinical isolates (SP1 and SP2) obtained from patient samples were identified using Gram staining and biochemical tests. As shown in (Figure 1), SP1 strain was Gram-positive, whereas strain SP2 was Gram-negative. Furthermore, additional biochemical tests revealed that all tested strains (SP1, *Staphylococcus aureus ATCC 29213* (*S. aureus* ST), SP2, and *Escherichia coli ATCC 25922* (*E. coli* ST)) were oxidase-negative and catalase-positive. Coagulase activity was detected only in SP1 and *S. aureus* ST, whereas both SP2 and *E. coli* ST exhibited negative coagulase reactions.

**Figure 2:**
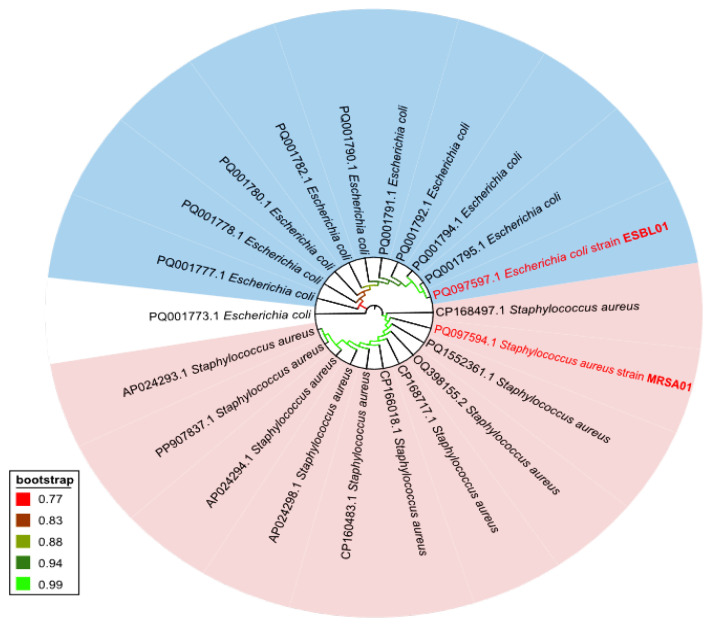
The phylogenetic tree for the 16S ribosomal RNA gene, the partial sequence for the pathogenic bacterial isolates under study (PQ097594 and PQ097597), and other sequences that are similar from blastn (http://blast.ncbi.nlm.nih.gov/Blast.cgi/, accessed on 7 October 2024). The phylogenetic tree was constructed by the MEGA 11 tool’s MUSCLE algorithm and neighbor-joining method with 1000 bootstrapping and visualized by iTOL. A red color label indicated our isolates, and the nodes were colored based on the bootstrap value. The tree is divided into clades, the blue one for *E. coli* sequences, which PQ097597 belongs to with a high bootstrap value (0.99). And the pink for *S. aureus* sequences, to which PQ097594 belongs with node has 0.99.

Based on *16S rRNA* gene sequencing and phylogenetic tree analysis (Figure 2), the SP1 isolate was identified as *Staphylococcus aureus* with a 491 bp sequence length and 100% similarity. This sequence has been deposited in the GenBank database under accession number PQ097594. The SP2 isolate was identified as *Escherichia coli* with a 553 bp sequence length and 100% similarity; its sequence was recorded in the same database under accession number PQ097597. Based on these findings, we designated SP1 strain as *Staphylococcus aureus* MRSA and SP2 strain as *Escherichia coli ESBL*.

### 2.2. Green Synthesis of Silver Nanoparticles

**Figure 3:**
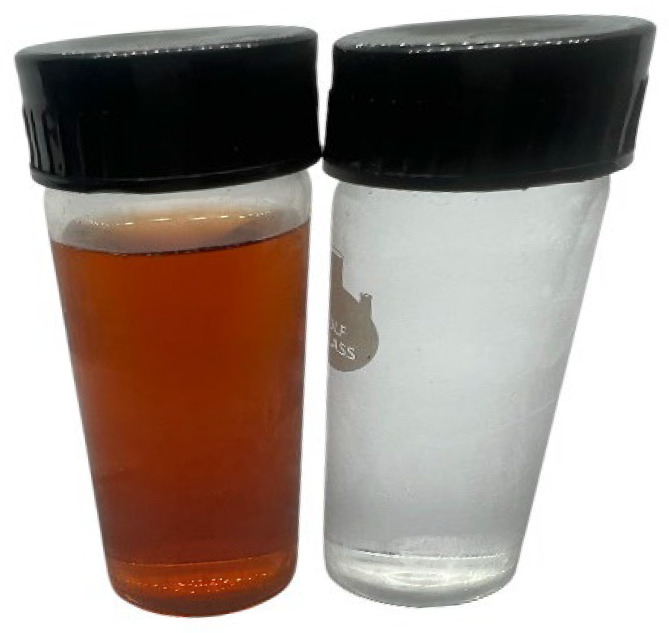
Green synthesis of silver nanoparticles with *C. arvensis* plant extract.

AgNPs were biosynthesized from *Convolvulus arvensis.* The ability of the plant extract’s active components to work as bioreductants for reducing silver ions to silver was investigated. After the addition of AgNO_3_, the color change to colloidal brown was investigated (Figure 3). This color signified the production of AgNPs The brown color indicates the formation of AgNPs due to surface plasmon resonance.

### 2.3. Characterization of Silver Nanoparticles

#### 2.3.1. UV Spectral Analysis

**Figure 4:**
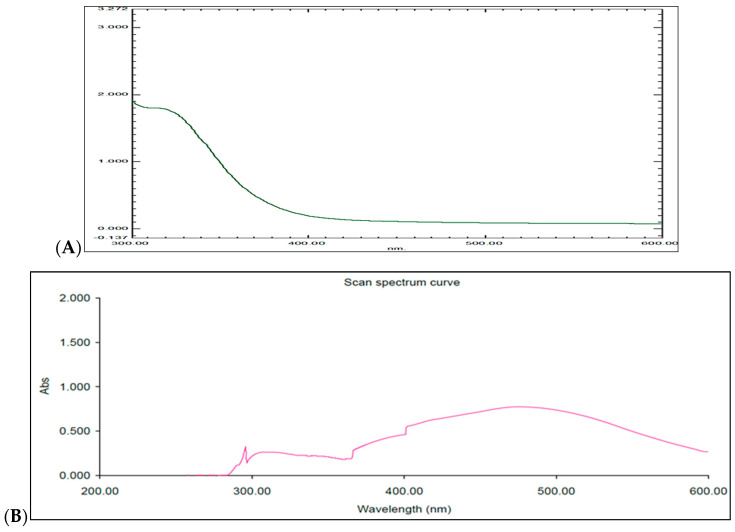
UV-Vis absorption spectrum. (**A**) *Convolvulus arvensis* aqueous extract (**B**) *Convolvulus arvensis* nanoparticles.

The absorption spectrum of the biosynthesized AgNPs was detected at 475 nm, indicating that there was no aggregation in the UV–Vis absorption spectrum (Figure 4). In comparison, the UV–Vis spectrum of the *Convolvulus arvensis* aqueous extract (Figure 4A) exhibited a broader and less intense absorption peak, indicating the presence of bioactive compounds but lacking the characteristic surface plasmon resonance of AgNPs. This comparison confirms the formation of well-dispersed silver nanoparticles and highlights the distinct spectral features of the biosynthesized AgNPs (Figure 4B) compared with the crude plant extract.

#### 2.3.2. Zeta Potential Determination and Particle Size Distribution

**Figure 5:**
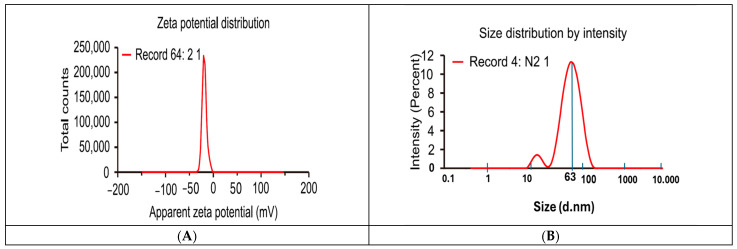
Characterization of AgNPs synthesized from *Convolvulus arvensis* extract (**A**) by Zeta potential determination and (**B**) Dynamic Light Scattering (DLS) displays the size distribution.

The surface charges of the *C. arvensis*-AgNPs were evaluated using zeta potential values (Figure 5A). A value of −18.9 mV was measured, signifying good stability for it. (Figure 5B) displays the hydrodynamic size distribution of the AgNPs as reported by Dynamic Light Scattering (DLS). It was found that the average size of AgNPs was 63 nm. And the polydispersity index (PdI) was 0.267.

#### 2.3.3. Scanning Electron Microscopy (SEM) Analysis

**Figure 6:**
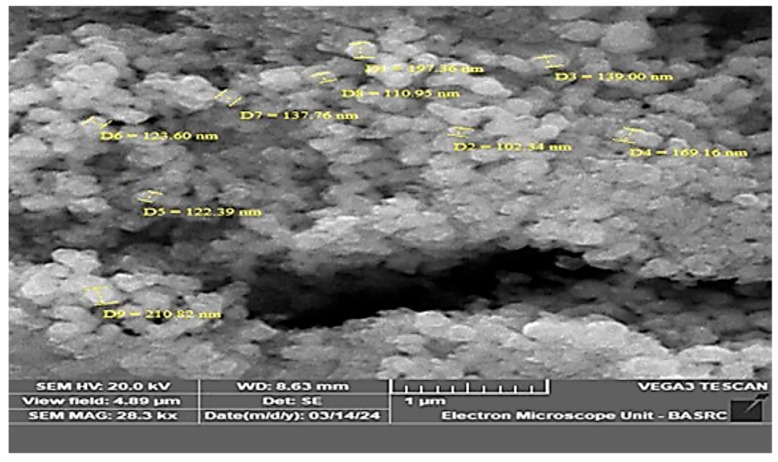
Scanning Electron Microscopy (SEM) image illustrating the spherical -shaped morphology of silver nanoparticles (AgNPs) synthesized using *Convolvulus arvensis* plant extract.

The Scanning Electron Microscopy (SEM) analysis showed that the silver nanoparticles synthesized using *C. arvensis* were well dispersed, spherical in shape, and ranged in size from 102.34 to 210.82 nm (Figure 6).

#### 2.3.4. Technical Specifications of EDX

**Figure 7:**
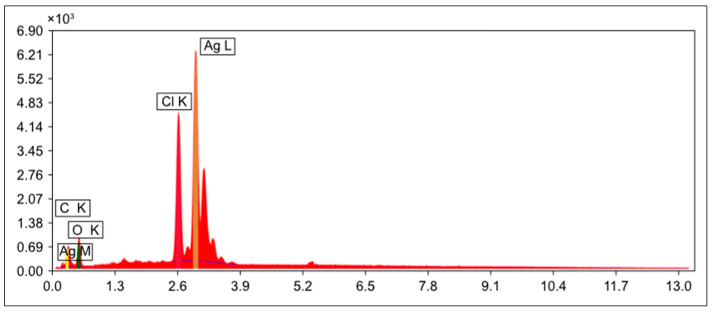
The elemental composition graph of AgNPs synthesized from *C. arvensis* extract by Energy-dispersive X-ray spectroscopy (EDX).

The EDX analysis confirmed the biosynthesis of AgNPs, as indicated by the strong silver signal. The elemental composition consisted of 60.15% silver, while oxygen, chloride, and carbon were detected at 16.69%, 16.36%, and 6.79%, respectively (Figure 7).

#### 2.3.5. Fourier-Transform Infrared Spectroscopy (FT-IR)

**Figure 8:**
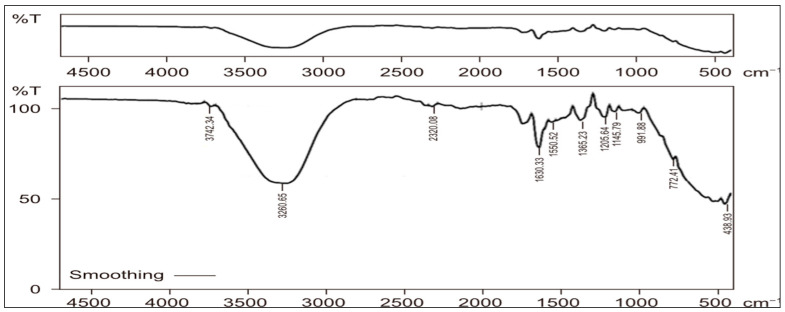
Characterization of AgNPs synthesized from *Convolvulus arvensis* extract by (FT-IR) Functional groups, including single, double, triple, and fingerprint bonds, were found.

The FTIR spectrum of the biosynthesized AgNPs is presented in (Figure 8), and the identified functional groups are summarized in (Table 1). The FTIR analysis of AgNPs synthesized using *C. arvensis* extract revealed several prominent absorption peaks at 3745.19, 3260.65, 2320.08, 1630.33, 1550.52, 1365.26, 1205.64, 1145.79, 991.88, 772.41, and 438.93 cm^−1^. These bands were assigned to O–H and N–H stretching vibrations, amino-related components, amide I and II groups, C–H bending, aromatic ring vibrations, C–N and C–O stretching modes, aliphatic phosphate groups, and sulfur-containing functional groups.

### 2.4. Estimation of Antibacterial Activity

#### 2.4.1. Antibacterial Bioassay Test

##### Diffusion Technique Assay

The results of the *C. arvensis*-AgNPs’ antibacterial tests against *S. aureus* MRSA, *S. aureus* ST, *E. coli* ST, and *E. coli* ESBL, using the agar disc diffusion technique displayed in (Table 2). The data revealed that *C. arvensis*-AgNPs had remarkable antibacterial activity against all bacterial strains used. *S. aureus* ST was most affected, followed by *E. coli ST,* then *E. coli ESBL*, and then *S. aureus MRSA*, with values of 13, 12, 11, and 10 mm, respectively.

##### Determination of Minimum Inhibitory Concentration (MIC) and Minimum Bactericidal Concentration (MBC)

The results of our investigation into the MIC and MBC are shown in (Table 3). The MIC for all strains was 12.5 µg/mL, except for *S. aureus ST,* in which it was 25 µg/mL; the MBC was 25 µg/mL, except for *S. aureus ST,* in which it was 50 µg/mL.

### 2.5. Evaluation of the Biological Effect of C. arvensis-Silver Nanoparticles

#### 2.5.1. Evaluation of Antibacterial Effects

**Figure 9:**
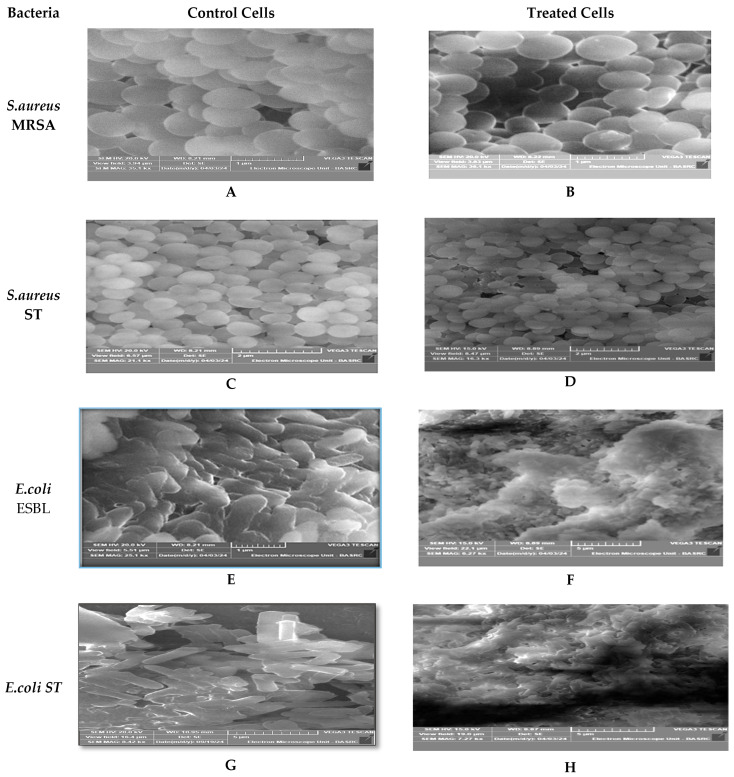
Scanning Electron Microscopy (SEM) images showing the morphological effects of silver nanoparticles synthesized from *C. arvensis.* (**A**–**C**) demonstrate the effect on Gram-positive *S. aureus* (MRSA and ST), E-H demonstrate the effect on Gram-negative *E. coli* ESBL and ST) bacterial strains. described above, and the cells exhibited a division-related septum, broadening, and cytoplasm leakage. Scanning electron microscope images showed that the untreated control groups (**A**,**C**,**E**,**G**), bacterial cells exhibited normal morphology with intact cell walls and smooth surfaces. In contrast, treated cells (**B**,**D**,**F**,**H**) showed significant morphological changes: *S. aureus* cells displayed shrinkage and deformations in their spherical shape, while *E. coli* cells exhibited marked structural disintegration and severe membrane damage. These observations confirm the destructive effects of silver nanoparticles on bacterial cell integrity. Most of the cells were damaged and perforated, which changed the cell structure and shape, and it was noted that some of them were empty. In addition, most cells appeared to stick together compared to untreated cells. As is clear in Figure 9.

An examination of the SEM micrographs of untreated cells (Figure 9) revealed a greater number of cells with intact structures and constant sizes, and a large extracellular matrix between them. After 2 h of culture with the AgNPs, we detected a significant decrease in the bacterial population compared to the control. Subsequent to treatment with *C. arvensis*-AgNPs (100 µg/mL), the presence of a septum was detected without cell division. However, this treatment led to the events.

#### 2.5.2. Determination of Antioxidant Activity

**Figure 10:**
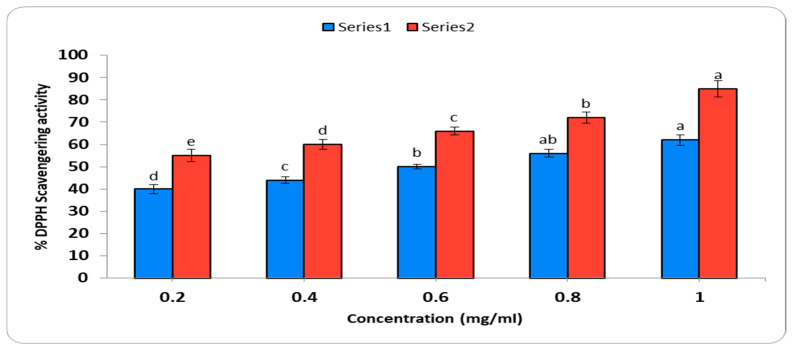
Determination of Antioxidant Activity: Estimation of DPPH radical scavenging activity from different concentrations of *C. arvensis*-AgNPs synthesized using *C. arvensis* leaf extract. Different letters indicate significant differences (*p* < 0.05) according to Duncan’s test.

Using gallic acid as a reference and based on shifts in color from violet to yellow, the DPPH radical scavenging activity of *C. arvensis*-AgNPs at different doses (0.2–1.0 mg/mL) was examined (Figure 10). At 1 mg/mL, gallic acid had the highest scavenging effectiveness (60.5%), indicating the strongest reducing action. It had an IC50 value of 0.15 mg/mL, and the half-maximal inhibitory concentration value was 0.71 mg/mL.

#### 2.5.3. Anti-Inflammatory Action

##### Suppression of Protein Denaturation

**Figure 11:**
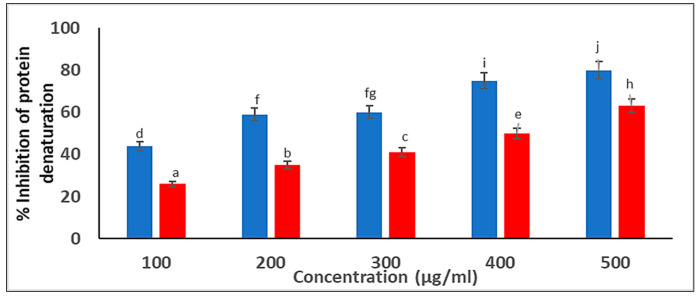
Anti-Inflammatory Action: Estimation of percentage inhibition of protein denaturation by *C. arvensis*-AgNPs. Different letters indicate significant differences (*p* < 0.05) according to Duncan’s test.

Protein denaturation inhibition is the chief mechanism of action of nonsteroidal anti-inflammatory drugs. Consequently, we determined the ability of the plant extract and biosynthesized *C. arvensis*-AgNPs to suppress protein denaturation. Both exerted a significant suppression of protein denaturation in a dose-dependent manner (Figure 11). At 500 μg/mL, the percentage suppression of protein denaturation was 69% and 55% for *C. arvensis*-AgNPs and the plant extract, respectively.

#### 2.5.4. Cytotoxicity Studies

**Figure 12:**
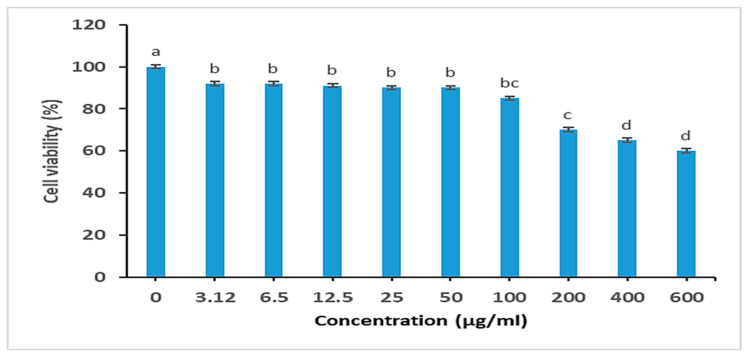
Cytotoxicity of *C. arvensis*-AgNPs in the MCF-7 cell line. The dose-dependent effect of *C. arvensis*-AgNPs was evaluated using an MTT assay after 24 h of treatment. Bars represent the standard error. Different letters indicate significant differences (*p* < 0.05) according to Duncan’s test.

The cell viability of *C. arvensis*-AgNPs was evaluated using an MTT assay on the MCF-7 cell line. As shown in (Figure 12), the *C. arvensis*-AgNPs exhibited a non-cytotoxic effect.

## 3. Discussion

Due to the global rise in antibiotic resistance, the need to discover novel antimicrobial agents has become more urgent than ever. In this study, silver nanoparticles (AgNPs) were green-synthesized using *Convolvulus arvensis* extract, and their biological efficacy was evaluated against multidrug-resistant (MDR) bacterial strains. The results demonstrate the promising therapeutic potential of these nanoparticles and provide a foundation for future in vivo investigations. The bacterial isolates-methicillin-resistant *Staphylococcus aureus* (MRSA) and extended-spectrum β-lactamase-producing *Escherichia coli* (ESBL) were identified using conventional biochemical tests and further confirmed by *16S rRNA* gene sequencing, a reliable method for rapid bacterial identification and species differentiation [30].

During the green synthesis of silver nanoparticles using *Convolvulus arvensis* extract, the color change from white or pale yellow to a colloidal brown indicated the formation of silver nanoparticles (AgNPs). This observation is consistent with previously reported studies [31,32,33].

The UV–Vis spectra of the biosynthesized silver nanoparticles (AgNPs) showed a characteristic surface plasmon resonance (SPR) peak at 475 nm, confirming the successful formation of well-dispersed nanoparticles. Compared to the crude aqueous extract of *Convolvulus arvensis*, the biosynthesized AgNPs in this study exhibited a more distinct and intense absorption peak, reflecting the unique optical properties arising from nanoparticle formation. Similar observations have been reported in previous studies employing plant-mediated green synthesis, such as the use of *Azadirachta indica* leaf extract, where an SPR peak around 420 nm was observed [34]. This variation in SPR peak position can be attributed to differences in particle size, morphology, and the nature of phytochemicals involved in the reduction and stabilization processes, which also aligns with previous studies highlighting the influence of particle size and distribution on SPR characteristics [31,32,35,36,37]. Additionally, the results support the role of biomolecules in stabilizing the nanoparticles, which likely contributes to their enhanced biological efficacy, including antimicrobial, antioxidant, and potential anti-inflammatory activities [37].

SEM analysis confirmed the presence of spherical nanoparticles, while EDX revealed that the elemental composition consisted of 60.15% silver, with the remaining elements, such as oxygen, chloride, and carbon, present in smaller percentages. Similar observations have been reported in previous studies [31,32,38].

FTIR analysis confirmed the presence of functional groups such as hydroxyl (–OH), C-O stretch, phenol, amide, thiols, aromatic ring and ether. These signals suggest the involvement of active phytochemicals including phenolics, flavonoids, and proteins in the reduction and stabilization of the AgNPs, validating previous observations by [39].

The AgNPs demonstrated antibacterial activity against *S. aureus* (MRSA and *ATCC 29213*) and *E. coli* (ESBL and *ATCC 25922*), with inhibition zones ranging from 10 to 13 mm. While slightly less potent than gentamicin (19 mm), the effect is still noteworthy. These observations support mechanisms previously proposed by [40], indicating that AgNPs disrupt bacterial membranes and trigger oxidative stress, thus limiting the potential for resistance development.

The synthesized AgNPs exhibited potent antibacterial activity, as evidenced by MIC and MBC values ranging from 12.5 to 25 µg/mL. These findings are in agreement with those reported by [10], who demonstrated comparable antimicrobial potency of *Convolvulus arvensis* extract-based nanoparticles against various bacterial strains, underscoring their effectiveness as alternative antimicrobial agents.

In comparison with MIC values of AgNPs synthesized from other plant sources, *C. arvensis* exhibited comparable or superior activity. For instance, refs. [10,41] reported MIC values ranging from 0.03 to 0.6 mg/mL using *Senna alexandrina*, underscoring the promising antibacterial potential of *C. arvensis*-derived nanoparticles.

SEM imaging of the treated bacteria showed significant damage to cell walls and membranes in both negative and positive Gram bacteria. The Ag^+^ ions released from the AgNPs attach to the microbial cell wall, inducing morphological alterations such as cytoplasm shrinking, membrane detaching, and cell wall rupturing [42]. These morphological changes are consistent with the findings reported in [18,31,38,43]. Moreover, the SEM examination showed a greater degree of structural involvement in Gram-negative bacteria. This observation aligns with [44], who reported that biosynthesized AgNPs are more effective against Gram-negative bacteria due to the peptidoglycan layer in their cell wall.

The *C. arvensis*-AgNp may have an effect on bacterial cell walls because it contains a hydroxyl group, as mentioned by [45], who said that incorporating hydroxyl (-OH) groups, particularly on the lipid A portion of lipopolysaccharide (LPS) in the outer membrane of Gram-negative bacteria, can adversely affect LPS structure and function.

Regarding antioxidant activity, the DPPH assay revealed a scavenging potential with an IC50 of 0.71 mg/mL, indicating a synergistic effect between the nanoparticle core and the plant-derived capping agents. These findings are consistent with previous studies by [46,47,48], which attributed antioxidant activity to the presence of flavonoids and polyphenolic compounds. Similarly, ref. [49] reported a high abundance of these bioactive constituents in *C. arvensis*.

In the current study, AgNPs synthesized from *Convolvulus arvensis* exhibited significant anti-inflammatory activity, particularly through their ability to inhibit protein denaturation, suggesting their potential utility in managing inflammatory conditions. These observations are consistent with those reported by [48,50], who found that AgNP-plant based extract showed antioxidant activity and reduction in inflammatory markers. Furthermore, our findings align with those of [51], who demonstrated that silver nanoparticles synthesized from *Cotyledon orbiculata* extract significantly reduced levels of pro-inflammatory cytokines such as TNF-α, IL-6, and IL-1β in LPS-stimulated macrophages. Collectively, these results highlight the therapeutic potential of biosynthesized AgNPs as effective anti-inflammatory agents.

Cytotoxicity testing on MCF-7 cells showed that the AgNPs were non-toxic at MIC levels, indicating a favorable initial safety profile. These findings are in agreement with those of [16,52], who reported that biosynthesized silver nanoparticles using *Moringa oleifera* extract exhibited selective cytotoxicity, showing minimal toxicity toward normal HUVEC cells while being more toxic to MCF-7 cancer cells. This suggests that plant-mediated AgNPs may offer biocompatibility advantages, consistent with our observations.

However, all evaluations were conducted in vitro, and further in vivo studies, dose–response analyses, and comprehensive toxicological assessments are required to confirm safety and therapeutic potential. Overall, C. arvensis–derived AgNPs show promise as alternative antimicrobial agents against antibiotic-resistant pathogens, warranting further investigation.

## 4. Materials and Methods

### 4.1. Bacterial Samples and Identification

#### 4.1.1. Bacterial Samples

Four bacterial strains were used in this study: one standard Gram-positive strain (*Staphylococcus aureus ATCC 29213*), one standard Gram-negative strain (*Escherichia coli ATCC 25922*), and two clinical isolates (SP1 and SP2) obtained from patient samples. All bacterial isolates were kindly provided by the microbiology laboratories at King Fahd University Hospital, Al Khobar, Saudi Arabia. The isolates were preserved in glycerol stocks and stored at 4 °C until further use.

#### 4.1.2. Identification of Bacteria

##### Gram Staining

Gram staining was performed using crystal violet solution, iodine solution, 99% ethanol, safranin solution, and distilled water. All reagents were obtained from Imam Abdulrahman Bin Faisal University, Saudi Arabia.

The standard Gram staining procedure was followed to identify and differentiate bacterial species. The process included bacterial culture preparation, incubation, staining, and microscopic examination using a light microscope (OLYMPUS, Tokyo, Japan). This staining method allows the classification of bacteria into two main groups based on their cell wall structure: (A) Gram-positive and (B) Gram-negative species [53].

##### Biochemical Tests

Biochemical identification of bacterial isolates was performed using the VITEK 2 system (BioMérieux, Marcy l′Etoile, France). This automated system enables rapid and accurate bacterial identification through direct inoculation from positive cultures, based on the analysis of biochemical reaction patterns [54].

##### *16S rRNA* Gene Sequencing and Phylogenetic Analysis

Genomic DNA for pathogenic bacteria isolates was extracted via a QIAamp DNA Mini Kit based on a column method (QIAGEN GmbH, Hilden, Germany). We quantified and purified the extracted DNA with a Nano-drop 2000c spectrophotometer (Thermo Scientific, Waltham, MA, USA). We used 2 primers targeting a segment of the *16S rRNA* gene, the forward 16S-27F primer (5′-AGAGTTTGATCMTGGCTCAG-3′), and the reverse 16S-800R primer (5′-TACCAGGGTATCTAATCC-3′). PCR conditions were as follows: 10 min of denaturation at 95 °C, 40 cycles of 30 s at 95 °C, 30 s at 55 °C, 30 s at 72 °C, 1 min at 72 °C, and a final 10 min at 72 °C. The amplification products were visualized on a 1.5% agarose gel and documented using a UV-based detection system, and the DNA fragment sizes were estimated using a 100-base pair (bp) DNA ladder. Then, we used ExoSAP-IT (Applied Biosystems, Thermo Fisher Scientific, Waltham, MA, USA) to purify amplicons. Sequence amplification was performed using the BigDye Terminator v3.1 Cycle Sequencing Kit (Thermo Fisher Scientific, USA) with 10 µM of either the forward (16S-27F) or reverse (16S-800R) primer. The DyeEx purification kit (QIAGEN, Germany) was used to purify sequencing reaction products, which were sequenced on the Seq Studio Genetic Analyzer (Applied Biosystems, USA). The sequences were analyzed using BLAST (http://blast.ncbi.nlm.nih.gov/Blast.cgi/, accessed on 7 February 2024) in order to find the most similar species; these were then submitted to GenBank. Following this, 10 nucleotide sequences, which were similar to each isolate’s *16s rRNA* sequence and produced using the BLAST tool [55,56,57] served as the basis for the phylogenetic trees. Then, the MEGA 11 tool’s MUSCLE algorithm [58], the neighbor-joining method [59,60], and a 1000-bootstrap value [61], were used for the phylogenetic tree, which was then visualized using iTOL (https://itol.embl.de/. accessed on 7 October 2024)

### 4.2. Collection of Plant Sample

The wild medicinal plant *Convolvulus arvensis* was obtained from the Department of Biology, College of Science, Imam Abdul Rahman bin Faisal University, Dammam, Saudi Arabia (GPS 26.407448625823644, 50.08418899191704). It was identified based on [62], as shown in (Table 4).

### 4.3. Green Synthesis of Silver Nanoparticles (AgNPs)

The fresh leaves and stem samples from *C. arvensis* were cut into 1 cm pieces, washed with distilled water, and then mixed with 10 g of plant sample with 100 mL of deionized distilled water. This solution was incubated in a water bath (Julabo, Seelbach, Germany) at 80 °C for 10 min. Then filtered by using a filter membrane.

In opaque containers, a 1:20 (*v*:*v*) ratio of aqueous leaf extract (at a concentration of 0.1 g/mL) to 1 mM AgNO_3_ was mixed and placed in a water bath at 60 °C for 20–30 min. A change in color from white or pale yellow to colloidal brown was observed within 20 min, indicating successful formation of silver nanoparticles. The containers were sealed and kept at 4 °C for further use. For characterization analysis, 100 mL of this nanoparticle solution was centrifuged at 4700 rpm for 20 min and then dried in an oven at 40 °C for subsequent experiments [31].

### 4.4. Characterization of Silver Nanoparticles

#### 4.4.1. UV Spectral Analysis

The reduction of silver ions was monitored using UV-Vis spectroscopy (UV-8400, Shimadzu, Kyoto, Japan). A 2 mL cuvette was filled with 1.5 mL of either the AgNPs solution or the corresponding aqueous plant extract for comparison. The absorbance was recorded over a wavelength range of 200–600 nm to characterize the AgNPs and to compare their spectral properties with the aqueous extract [31].

#### 4.4.2. Zeta Potential Determination and Particle Size Distribution

Zeta potential can be informative in predicting nanoparticle effects; we measured these values using a spectrophotometer from Malvern Analytical (Malvern Panalytical Ltd, Malvern, UK). To find out the hydrodynamic size and polydispersity index of the produced nanoparticles, we employed dynamic light scattering (DLS). Zeta potential measurements are crucial for determining whether AgNPs are stable in aqueous solutions. AgNPs usually exhibit good stability when their zeta potential is less than −25 mV or larger than +25 mV [31,37,38].

#### 4.4.3. Scanning Electron Microscopy (SEM)

We also used scanning electron microscopy (SEM) alone to screen AgNPs and determine their product structure, as well as AgNP biosynthesis. The sample was mounted on the metallic stub and fixed using double-faced adhesive carbon. The stubs were coated three times using sputtering, and the samples were then visualized. The SEM had the following technical specifications: the use of a scanning electron microscope Tescan VEGA3 SEM (TESCAN ORSAY HOLDING a.s., VEGA3, Brno, Czech Republic) (Tescan, Brno, Czech Republic) with a detector for secondary electrons (SEs), BSE, and LVST. The chamber’s internal size is 160 mm, and the maximum specimen height is 36/34 [31].

#### 4.4.4. Technical Energy-Dispersive X-Ray Spectroscopy (EDX) Specifications

This method is used to evaluate the elemental composition of materials. The sample was inserted into the scanning electron microscope equipped with an energy-dispersive X-ray spectroscopy (EDX) device. This helps us measure the racial composition of the sample, facilitating our understanding and description of the elements’ properties across diverse applications. EDX is a widely used technique for studying elemental composition and distribution in nanomaterials. It involves high-energy electron bombardment, resulting in the release of X-ray photons. These X-rays can be categorized by element and represented in a spectrum or mapping [31].

#### 4.4.5. Fourier-Transform Infrared Spectroscopy (FT-IR)

We characterized AgNPs for biological samples using Fourier-transform infrared spectroscopy (FTIR-8400, Shimadzu, Japan). The FTIR measurements ranged from 500 to 4500 cm to identify the functional groups of the AgNPs [29,31].

### 4.5. Evaluation of the Biological Effect of Silver Nanoparticles Synthesized

#### 4.5.1. Antibacterial Bioassay Test

##### Diffusion Technique Assay

Agar well diffusion was used to measure antimicrobial activity. We placed 0.5 mL of a microorganism culture, aged 18 to 24 h (standard inoculum 1.5 × 108 CFU/mL 0.5 McFarland standard), in Petri dishes, and 15 mL of nutrient agar was added to the plates. Using a sterile cork borer, 5 mm wells were punched once the cultures had hardened. Then, 50 µL of AgNPs was added to each well; gentamicin (GM 30 µg) served as a positive control, and AgNO3 as a negative control. For one hour, the treated plates were refrigerated to facilitate AgNPs and control diffusion. After that, the plates were incubated at 37 °C for 18 to 24 h. By testing the antimicrobial activity, the zones of inhibition around the wells were measured in millimeters. Larger inhibition zones indicated stronger antibacterial activity. All experiments were performed in triplicate [31].

##### Determination of Minimum Inhibitory Concentration (MIC)

The MIC is the lowest concentration of an antimicrobial agent that can, after overnight incubation, inhibit the visible growth of a microorganism. They were performed in triplicate [31].

##### Determination of Minimum Bactericidal Concentration (MBC)

In determining the MBC, we used the pouring plate method. After performing the MIC experiment, the contents were transferred to a Petri dish, and the liquefied medium was poured over it and mixed well. They were then transferred to an incubator at 37 °C for 24 h before the results were recorded. The plates on which no bacterial colonies appeared represented the MBCs. All experiments were performed in triplicate [31].

#### 4.5.2. Evaluation of Antibacterial Effects by Scanning Electron Microscope (SEM)

SEM analysis was used to examine the morphological changes on the surface of the bacteria. Microbial cultures were grown in nutrient broth (NB). The cells were incubated at 37 °C overnight, fixed with 10% formaldehyde, then dehydrated with serial methanol and air dried, and were performed in triplicate. These were then examined using a Tescan VEGA3 SEM (TESCAN ORSAY HOLDING a.s., VEGA3, Brno, Czech Republic) (Tescan, Brno, Czech Republic) [31,63].

#### 4.5.3. Determination of Antioxidant Activity

Using the DPPH radical scavenging experiment, the AgNPs were utilized to quantify the antioxidant function [64]. Using gallic acid as the standard, the decrease in DPPH was spectrophotometrically measured at 517 nm in comparison to the blank.

The percentage of inhibition was calculated using Equation (1).

(1) $$\%\;inhibition=\frac{\left(A_{blank}-A_{sample}\right)}{100}\times 100\%$$

The percentage inhibition of the sample was determined using the following equation:

%Inhibition = A_blank_ − A_sample_A_blank_ × 100%

where A_blank_ represents the absorbance of the blank/control and A_sample_ represents the absorbance of the test sample.

And then the linear plot of % inhibition versus concentration was analyzed (Equation (2)).

Y = a + bx(2)

where x is the concentration of the measured substance, and y is the % inhibition. Meanwhile, the IC50 value was determined as the x value of this equation when y was equal to 50% and was in triplicate [38].

#### 4.5.4. Anti-Inflammatory Activity of the Biosynthesized AgNPs

##### Suppression of Protein Denaturation

Protein suppression was utilized to assess the anti-inflammatory activity of the *C. arvensis* extract and the biosynthesized AgNPs, following the method described by Aldayel [38]. An egg solution was added to 2.5 mL of PBS and 2 mL of *C. arvensis* leaf extract. The mixtures were incubated at 37 °C before being heated to 70 °C. A spectrophotometer set at 660 nm was used to measure the turbidity. The percentage of suppressed protein denaturation was ascertained as follows:

Percentage of suppression of protein denaturation = 100 × [1 − Absorbance (sample)/Absorbance (Control)].

All experiments were performed in triplicate.

#### 4.5.5. Cytotoxicity Study

##### Cell Culture

The MCF-7 cell line was donated by the Leibniz Institute DSMZ German Collection of Microorganisms and Cell Cultures.

##### Cytotoxicity Study

Using an MTT cell proliferation test kit to measure cell viability, the cytotoxic activity of the AgNPs was determined. After being cultured on plates, different concentrations of AgNPs were incubated with the cells for 48 h. The cells were washed in PBS before being cultured in fresh medium containing MTT. Utilizing an ELISA plate reader (BMG LABTECH GmbH (Ortenberg, Germany) the optical density was measured at 550 nm [38] and performed in triplicate.

### 4.6. Statistical Analysis

SPSS 2007 (Ver. 17.0) was used for ANOVA calculations in order to investigate the ability of plant nanoparticles to inhibit selected bacterial strains. The significance value was measured at *p* ≤ 0.01.

## 5. Conclusions

Silver nanoparticles synthesized using *Convolvulus arvensis* (*C. arvensis*-AgNPs) demonstrated significant antibacterial activity against two multidrug-resistant clinical pathogenic isolates, SP1 and SP2. These isolates were identified by biochemical tests and *16S rRNA* gene sequencing as *Staphylococcus aureus* MRSA (accession number PQ097594) and *Escherichia coli* ESBL (accession number PQ097597), respectively. The *C. arvensis*-AgNPs also exhibited antibacterial activity against the standard pathogenic strains *Staphylococcus aureus* ATCC 29213 and *Escherichia coli* ATCC 25922.

In addition to their antimicrobial properties, *C. arvensis*-AgNPs exhibited prominent antioxidant and anti-inflammatory activities, with no cytotoxic effects observed on MCF-7 cells at the tested concentrations. These findings suggest that biosynthesized AgNPs derived from *C. arvensis* possess multifunctional biological efficacy.

Further investigation of *C. arvensis*-derived AgNPs is recommended for potential pharmaceutical and food-related applications due to their safety profile and multifunctional properties. Moreover, future studies should focus on identifying the specific bioactive compounds responsible for these effects and evaluating their potential biomedical applications.

## Figures and Tables

**Table 1:** Recorded FTIR wavenumbers and corresponding band assignments of *C. arvensis*–AgNPs.

Wavenumber (cm^−1^)	Assigned Functional Group	Interpretation	Reference
~3724	O–H stretching	Free hydroxyl groups of alcohols or phenols	[26]
~3260	O–H/N–H stretching	Hydrogen-bonded hydroxyl groups or amine groups	[26]
~2350	N–H component	Amino-related component	[27]
~1630	Open-chain azo (-N=N-)	Amide I	[26]
~1556	N–H bending	Amide II band, Carboxylate (carboxylic acid salt) Amide	[26]
~1452	C–H bending, C=C-C Aromatic ring stretch	deformation of –CH_2_	[26,28]
~1360	C–N stretching, deformation of O–H	gem-Dimethyl or Trimethyl, Aliphatic nitro compounds	[26,28]
~1200	C–O stretching	Phenols, Organic sulfates	[26]
~1140	Cyclic ethers, C-O stretch	Ether and oxy compound	[29]
~990	Aliphatic phosphates (P-O-C stretch)	Simple hetero-oxy compounds	[26]
~770	C-H Monosubstitution (phenyl), C-H 1,2-Disubstitution (ortho)	Aromatic ring (aryl)	[26]
~430	Aryl disulfides (S-S stretch)	Thiols and thio-substituted compounds	[26,29]

**Table 2:** Antibacterial potential of *C. arvensis*-AgNPs against some pathogenic bacteria. (Antibacterial tests against *S. aureus* MRSA, *S. aureus ST*, *E. coli ST*, and *E. coli ESBL*, using the agar disc diffusion technique).

Bacteria	Gram-Positive Bacteria		Gram-Negative Bacteria	
	*S. aureus* MRSA	*S. aureus* ST	*E. coli* ESBL	*E. coli* ST
	Zone of Inhibition (mm) ± Standard Deviation			
*C. arvensis* plant nanoparticles	10 ± 0.00	13 ± 0.33	11.33 ± 0.33	11.66 ± 0.887
Gentamycin	10 ± 0.00	10 ± 0.00	19 ± 0.33	19 ± 0.33
Significance (*p* ≤ 0.01)	0.12	0.020	0.001	0.010

**Table 3:** Minimal inhibitory concentration (MIC) µg/mL and minimal bactericidal concentration (MBC) µg/mL ± Standard Deviation of *C. arvensis* plant nanoparticles.

Test Bacteria	MIC µg/mL	MBC µg/mL
	Concentration ± Standard Deviation	
*S. aureus* MRSA	12.5 ± 0.00	25 ± 0.00
*S. aureus* ST	25 ± 0.00	50 ± 0.00
*E. coli* ESBL	12.5 ± 0.00	25 ± 0.00
*E. coli* ST	12.5 ± 0.00	25 ± 0.00

**Table 4:** *Convolvulus arvensis* information.

Name	Date of Collection	Part of Plant	Picture	Site
*Convolvulus* *arvensis*	15 March 2023	Stem and leaves	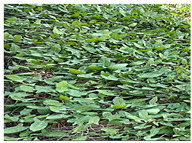	26.407448625823644, 50.08418899191704

## Data Availability

The original contributions presented in this study are included in the article. Further inquiries can be directed to the corresponding author.

## Abbreviations

The following abbreviations are used in this manuscript:

AgNPs	Silver nanoparticles
SEM	Scanning electron microscopy
EDX	Energy-dispersive X-ray spectroscopy
FTIR	Fourier-transform infrared spectroscopy
MIC	Minimum inhibitory concentration
MBC	Minimum bactericidal concentration
NCBI	National Institutes of Health
